# Novel approaches to develop Rift Valley fever vaccines

**DOI:** 10.3389/fcimb.2012.00131

**Published:** 2012-10-26

**Authors:** Sabarish V. Indran, Tetsuro Ikegami

**Affiliations:** ^1^Department of Pathology, The University of Texas Medical BranchGalveston, TX, USA; ^2^Sealy Center for Vaccine Development, The University of Texas Medical BranchGalveston, TX, USA; ^3^Center for Biodefense and Emerging Infectious Diseases, The University of Texas Medical BranchGalveston, TX, USA

**Keywords:** Rift Valley fever virus, bunyavirus, phlebovirus, vaccine, replicon, vaccine vector, subunit vaccine

## Abstract

Rift Valley fever (RVF) is endemic to sub-Saharan Africa, and has spread into Madagascar, Egypt, Saudi Arabia, and Yemen. Rift Valley fever virus (RVFV) of the family *Bunyaviridae*, genus *Phlebovirus* causes hemorrhagic fever, neurological disorders or blindness in humans, and high rate abortion and fetal malformation in ruminants. RVFV is classified as a Category A Priority pathogen and overlap select agent by CDC/USDA due to its potential impact on public health and agriculture. There is a gap in the safety and immunogenicity in traditional RVF vaccines; the formalin-inactivated RVFV vaccine TSI-GSD-200 requires three doses for protection, and the live-attenuated Smithburn vaccine has a risk to cause abortion and fetal malformation in pregnant ruminants. In this review, problems of traditional vaccines and the safety and efficacy of recently reported novel RVF candidate vaccines including subunit vaccines, virus vector, and replicons are discussed.

Rift Valley fever (RVF) is a mosquito-borne zoonotic disease endemic to sub-Saharan Africa, and has spread into Egypt, Madagascar, and the Arabian peninsula (Swanepoel and Coetzer, 2004). RVF is characterized by high rate of abortion or fetal malformation in ruminants, and hemorrhagic fever, neurological disorder, or blindness in humans (Ikegami and Makino, 2011). Rift Valley fever virus (RVFV: family *Bunyaviridae*, genus *Phlebovirus*) has a tripartite negative-stranded RNA genome: S-, M-, and L-segments. S-segment encodes the N and NSs genes in an ambisense manner, M-segment encodes the Gn, Gc, 78 kD and NSm genes, and L-segment encodes the L gene (Ikegami, 2012). NSs and NSm are non-structural proteins, and NSs is a major virulence factor of RVFV (Bouloy et al., 2001). RVFV is classified as a Category A Priority Pathogen by NIAID/NIH, and overlap select agent by US Department of Health and Human Services (HHS) and Agriculture (USDA). Only vaccination can effectively prevent the spread of RVFV, and the development of live-attenuated, inactivated or other available vaccines for RVF was reviewed previously (Ikegami and Makino, 2009). Here, we review the development of novel candidate vaccines for RVF in recent years such as subunit vaccines, non-spreading RVFV replicons, alphavirus replicons, adenovirus replicons, poxvirus vector, and Newcastle virus vector.

## Traditional strategies

There is a gap between safety and immunogenicity for RVF vaccine development; (1) live-attenuated vaccines are immunogenic and induce protective immunity by a single dose while lacking safety due to a potential of reversion to virulence or spread into environment, (2) formalin-inactivated vaccines are safe in animals and humans, while lacking immunogenicity to induce sufficient neutralizing antibody by a single dose.

The live-attenuated MP-12 strain was developed by 12 serial mutagenesis of wild-type (wt) ZH548 strain by 5-fluorouracil (Caplen et al., 1985), and encodes virulent S-segment and attenuated M- and L-segments (Billecocq et al., 2008). MP-12 is safe and immunogenic in ruminants and humans, and is excluded from the select agent rule in the US. The attenuation of MP-12 occurs by point mutations, which raises two concerns; (1) a potential of reversion to virulence and (2) a lack of DIVA marker (differentiating infected from vaccinated animals). In addition, a past study suggested that MP-12 causes abortion and fetal malformation in ewes when vaccinated at an early stage of pregnancy (Hunter et al., 2002). Thus, further improvement of MP-12 is warranted. Two segmented recombinant MP-12 (r2segMP12) encoding Gn and Gc (nt.411 to 3614 of M-segment) in place of NSs, and lacking M-segment was created and improved safety by reducing the risk of potential reassortment with wt RVFV (Brennan et al., 2011). Lihoradova et al. showed that a recombinant MP-12 lacking NSs is highly efficacious and useful for DIVA in a mouse model (Lihoradova et al., 2012). The efficacy of those improved MP-12 in ruminants and non-human primates are yet to be determined. On the other hand, live-attenuated candidate vaccines derived from wt RVFV strains other than ZH548 have been developed for veterinary use. A natural plaque isolate from 74HB59 strain, named the Clone 13 strain, encodes a 69% in-frame truncation in the NSs gene (Muller et al., 1995), and is completely attenuated in animals due to a lack of functional NSs, regardless of the wt M- and L-segments (Bouloy et al., 2001). Clone 13 was safe in ewes at 15, 50, and 100 days of pregnancy, while efficacious with at least 10^5^ pfu per dose (Dungu et al., 2010). Clone 13 induced neutralizing antibodies by day 7 without developing viremia in cattle (von Teichman et al., 2011). The recombinant ZH501 strain lacking both NSs and NSm genes (ΔNSs-ΔNSm rRVFV vaccine) also safely induced protective immunity in ewes at 42 days of pregnancy (Bird et al., 2011). Those vaccines encode a large deletion of RVFV gene(s), and are useful for DIVA with a minimum concern of reversion to virulence. However, those candidate vaccines are classified as select agents in the US.

The formalin-inactivated Salk Institute-Government Services Division (TSI-GSD)-200 vaccine was developed by using diploid rhesus lung cell line (FRhL-2, DBS 103 cells) (Kark et al., 1982). Among 598 vaccinees, TSI-GSD-200 vaccine elicited Plaque Reduction Neutralizing Test: PRNT_80_ 1:40 or more in 90.3% after three doses (s.c.), while PRNT_80_ 1:40 was maintained in 50% of vaccines only for 287 days without subsequent boosters (Pittman et al., 2000).

## Subunit vaccines

Preparation of inactivated RVFV requires high containment facilities. Also, use of chemicals such as formalin may modify RVFV epitopes. Efficacy of highly purified Gn ectodomain was tested in mice (de Boer et al., 2010). Gn ectodomain (Gn-e) lacking 109 aa. of Gn C-terminus was expressed with C-terminus His-tag in *Drosophila* Schneider (S2) cells using an insect expression vector encoding an inducible metallothionein promoter and *Drosophila* BiP secretion signal. Mice vaccinated intraperitoneally twice with Gn-e with Stimune (a water-in-oil adjuvant) induced neutralizing antibodies after the second dose and survived challenge with RVFV M35/74 strain (South African strain) (Table 1). In a later study, the Gn-e was fused with Flag-tag, enterokinase cleavage site and three Strep-tags, and purified from *Drosophila* S2 cells with Strep-Tactin Sepharose (Kortekaas et al., 2012). The purified Gn-e was named GneS3, and formulated in Stimune adjuvant. Lambs subcutaneously vaccinated once with GneS3 vaccine induced neutralizing antibodies in four out of six lambs at 19 days post vaccination, and were protected from viramia and fever after challenge with recombinant M35/74 strain (Table 1). On the other hand, mild to moderate swelling of the injection sites were observed in vaccinated lambs. The purified Gn ectodomain will be useful as human RVF vaccine because it is superior to the TSI-GSD-200 vaccine in the large scale production without a risk of infection.

**Table 1:** **Recent development of RVF vaccines**.

**Vaccine type**	**Vector**	**Name**	**RVFV antigen**	**Animal**	**Dose^*a*^**	**Booster^*b*^**	**Challenge^*c*^**	**Efficacy^*d*^**	**References**
Subunit	−	Gn-e	Gn ectodomain	Balb/c mice	10 μ g (i.p.)	10 μ g (i.p.) (day 21)	M35/74 (day 42) 10^2.7^ TCID_50_ (i.p.)	100% (survival)	de Boer et al., 2010
	−	GneS3	Gn ectodomain	Lambs	20 μ g (s.c.)	−	rec M35/74 (day 19) 10^5^ TCID_50_ (i.p.)	100% (viremia, clinical sign)	Kortekaas et al., 2012
Poxvirus vector	VACV Copenhagen strain	vCOGnGc	Gn and Gc	CB6F_1_ mice	10^7^ pfu (i.m.)	−	ZH501 (22 weeks) 10^3^ pfu (i.p.)	50% (survival)	Papin et al., 2011
				CB6F_1_ mice	10^7^ pfu (i.m.)	10^7^ pfu (i.m.) (8 weeks)	ZH501 (22 weeks) 10^3^ pfu (i.p.)	90% (survival)	Papin et al., 2011
				Baboon	10^7^ pfu (scarification)	10^9^ pfu (i.m.) (day 28)	NA	NA	Papin et al., 2011
	LSDV KS1 strain	rLSDV-RVFV	Gn and Gc	Sheep	10^6^ or 10^7^ pfu (i.d.)	10^6^ or 10^7^ pfu (i.d.) (day 21)	AR 20368 (5 weeks) 10^7^ pfu (i.m.)	100% (fever)	Wallace et al., 2006
	LSDV KS1 strain	rKS1/RVFV	Gn and Gc	Sheep	10^7^ TCID_50_ (s.c.)	10^7^ TCID_50_ (s.c.) (3 weeks)	Smithburn (7 weeks) 10^7^ TCID_50_ (s.c.)	100% (viremia, fever)	Soi et al., 2010
NDV vector	NDV LaSota strain	NDFL - Gn	Gn	Cattle	2 × 10^7^ TCID_50_ (i.m.)	2 × 10^7^ TCID_50_ (i.m.) (day 28)	NA	NA	Kortekaas et al., 2010a
	NDV LaSota strain	NDFL - GnGc	Gn and Gc	Balb/c mice	10^5.3^ TCID_50_ (i.m.)	10^5.3^ TCID_50_ (i.m.) (day 21)	rec M35/74 (day 42) 10^2.7^ TCID_50_ (i.p.)	100% (survival)	Kortekaas et al., 2010b
				Lambs	10^7^ TCID_50_ (i.m.)	−	rec M35/74 (day 19) 10^5^ TCID_50_ (i.p.)	100% (viremia, clinical sign)	Kortekaas et al., 2012
AdV replicon	Ad5	CAdVax-RVF	Gn and Gc	CD1 mice	10^8^ pfu (i.p.)	−	ZH501 (11 weeks) 10^2^ pfu (i.p.)	100% (survival)	Holman et al., 2009
				CD1 mice	10^8^ pfu (i.p.)	10^8^ pfu (i.p.) (day 21)	ZH501 (27 weeks) 10^2^ pfu (i.p.)	100% (survival)	Holman et al., 2009
Alphavirus replicon	VEEV	VEErep/Gn	Gn	Balb/c mice	10^6^ pfu (s.c.)	−	ZH501 (day 42) 10^3^ pfu (s.c.)	100% (survival)	Gorchakov et al., 2007
	SINV AR86 isolate	REP91-RVF(M)	NSm, Gn, and Gc	NIH Swiss mice	10^5^ pfu (footpad)	10^5^ pfu (footpad) (4 weeks)	VRL 688/78 (7 weeks) 10^2^ mouse LD_50_ (i.p or i.n.)	100% (survival)	Heise et al., 2009
	SINV Girdwood isolate	Rgrid-RVF(M)	NSm, Gn, and Gc	NIH Swiss mice	10^5^ pfu (footpad)	10^5^ pfu (footpad) (4 weeks)	VRL 688/78 (7 weeks) 10^2^ mouse LD_50_ (i.p or i.n.)	100% (survival)	Heise et al., 2009
RVFV replicon	RVFV M35/74 strain	RRP (NSR)	Gn, Gc, N, and L	Balb/c mice	10^7^ TCID_50_ (i.m.)	−	rec M35/74 (day 42) 10^2.7^ TCID_50_ (i.p.)	100% (survival)	Kortekaas et al., 2011
				Balb/c mice	10^7^ TCID_50_ (i.m.)	10^7^ TCID_50_ (i.m.) (day 21)	rec M35/74 (day 42) 10^2.7^ TCID_50_ (i.p.)	100% (survival)	Kortekaas et al., 2011
				Sheep	10^7^ TCID_50_ (i.m.)	−	rec M35/74 (day 19) 10^5^ TCID_50_ (i.p.)	100% (viremia, clinical sign)	Kortekaas et al., 2012
	RVFV ZH501 strain	VRP_RVF_	Gn, Gc, N, and L	C57BL/6 mice	10^4^ or 10^5^ TCID_50_ (s.c.)	−	ZH501 (day 28) 10^5^ pfu (s.c.)	100% (survival)	Dodd et al., 2012

a Vaccine dose and inoculation route, i.p., intraperitoneal; s.c., subcutaneous; i.m., intramuscular; i.d., intradermal.

b Booster dose, route, and timing.

c RVFV strain for challenge, timing, dose, and route.

d Efficacy and evaluation of protection.

NDV, Newcastle disease virus; AdV, Adenovirus; NA, not available.

## Non-spreading RVFV replicons

Recently, using reverse genetics, RVFV itself has been modified into non-spreading replicon for vaccine use. Kortekaas et al. generated reverse genetics system for M35/74 strain and used it for production of replicons which can infect mammalian cells but cannot spread to neighboring cells (Kortekaas et al., 2011). BHK cells infected with fowlpoxvirus encoding T7 RNA polymerase were transfected with plasmids encoding RVFV genome S-GFP (NSs was replaced with GFP) and L as well as plasmid encoding M ORF. The resulting RVFV replicon particles (RRPs) in the supernatants yielded up to 1 × 10^4.8^ TCID_50_. RRPs could be amplified up to 1 × 10^6.7^ TCID_50_ in BHK cells or 293 cells infected with RRPs with the co-transfection of plasmids encoding Gn, Gc, and/or NSm. GFP-positive cells were increased after serial passages or clonal selection using Geneticin. The resulting replicon cell line did not contain detectable fowlpox DNA, while S- and L-segment RNA encoded a few mutations. Furthermore, the passage of replicon cells in the presence of neutralizing serum did not result in a decrease in the number of GFP-positive cells. Mice were vaccinated once or twice (i.m. or s.c.) with 1 × 10^6^ TCID_50_ of RRPs and challenged with M35/74 at day 42. The survival rate was 60, 60, 100 or 100% by s.c. (x1), s.c. (x2), i.m. (x1) or i.m. (x2), respectively (Table 1). Sheep were vaccinated once (i.m.) with 1 × 10^7^ TCID_50_ of RRP (non-spreading RVFV: NSR) and challenged with M35/74 at day 19 post vaccination (Kortekaas et al., 2012). All vaccinated sheep developed neutralizing antibodies, and were protected from viremia or illness after wt RVFV challenge (Table 1). Dodd et al. generated ZH501 replicons by a different approach in which BSR-T7/5 cells, which stably express T7 RNA polymerase, were transfected with plasmids encoding genome S (NSs is replaced with GFP) and L as well as plasmid encoding the ORF of Gn and Gc (Dodd et al., 2012). Virus replicon particles (VRP_RVF_) were directly collected from the supernatants with 100% efficiency with titers ranging from 1 × 10^6^ to 5 × 10^7^ TCID_50_/ml. All newborn suckling mice (2-day-old) inoculated (i.c.) with 1 × 10^4^ or 1 × 10^5^ TCID_50_ of VRP_RVF_ survived. Mice vaccinated once (s.c.) with VRP_RVF_ and challenged with ZH501 also survived (Table 1). Furthermore, 80% protection was achieved with 1 × 10^4^ or 1 × 10^5^ of VRP_RVF_ by 2 days post-vaccination. Those RVFV replicons are highly safe and immunogenic, and will be suitable for use in both humans and animals. Further improvements in process development will be required to scale-up the production in certified cell substrates for industrial manufacture.

## Alphavirus replicons

Alphaviruses (family *Togaviridae*), a member of positive-stranded RNA viruses, have been used as replicons by expressing foreign gene in place of viral structural genes. Since structural proteins are not expressed in replicon particles, antivector responses are generally minimal (Rayner et al., 2002). Several alphavirus replicons expressing RVFV NSm, Gn and/or Gc using Sindbis (SIN) replicons and Venezuelan equine encephalitis virus (VEEV) replicon (VEErep/Gn) were characterized (Gorchakov et al., 2007). SIN replicons did not induce protection in mice, whereas all mice vaccinated once (s.c.) with 1 × 10^6^ pfu of VEErep/Gn and challenged with ZH501 survived (Table 1) even with neutralizing antibody titer below 1:80 before challenge. However, the VEEVrep/Gn could not be passaged in cell culture due to strong interference of Gn with VEE replication. It could be due to resistance to type-I IFN in cells supporting VEEV replication. Two types of SIN replicons; REP91-RVF(M) and Rgrid-RVF(M) were generated from AR86 and Girdwood isolates from South Africa, respectively (Heise et al., 2009). Mice vaccinated twice (footpad) with 1 × 10^5^ IU of REP91-RVF(M) or Rgrid-RVF(M) and challenged with wt RVFV all survived without overt signs of illness (Table 1). Prime-boost vaccination using alphavirus replicon was tested in a mouse model (Bhardwaj et al., 2010). Mice were vaccinated twice (gene gun at 0 and 3 weeks) with 2 μ g of DNA encoding RVFV Gn ectodomain (aa.131–557) fused to tandem repeats of mouse homolog of C3d, and with VEE encoding the same Gn ectodomain at 1 × 10^5^ IU (footpad at 6 weeks). All mice survived from ZH501 challenge, and induced mixed Th1/Th2 response, while mice vaccinated with DNA only (Gn-C3d) at 0, 3, and 6 weeks also survived from lethal challenge but induced Th2-restricted immune response. Thus, alphavirus-based vaccine will be applicable to RVF vaccines for both human and veterinary uses.

## Adenovirus replicons

Adenovirus replicons are widely used for vaccine or gene therapy, and accommodate large size (up to 37 kb) of foreign DNA (Goncalves and de Vries, 2006). A complex adenovirus (CAdVax) vector system is based on a modified Ad5sub360 vector backbone with deletions in E1, E3, and almost all E4 ORFs with the exception of ORF6 (Wang et al., 2006). Holman et al. developed CAdVax encoding RVFV Gn and Gc (codon optimized for humans) of MP-12 strain with human CD4 signal peptides under human cytomegalovirus (CMV) promoter, designated as CAdVax-RVF (Wang et al., 2006; Holman et al., 2009) for human RVF vaccine. All CD1 mice vaccinated once or twice (i.p.) with 1 × 10^8^ pfu of CAdVax-RVF and challenged with ZH501 strain at 11 or 27 weeks, respectively, survived (Table 1) (Holman et al., 2009). To test the influence of pre-existing immunity against adenovirus replicon, mice were immunized with 1 × 10^9^ pfu of CAdVax-D encoding dengue virus glycoprotein, and subsequently vaccinated with 1 × 10^6^ (low dose) or 1 × 10^8^ pfu (high dose) of CAdVax-RVF 10 weeks later. Although the antibody level was limited in low dose group, the high dose group showed high levels of anti-Gn/Gc antibody responses. On the other hand, only 25% of mice with a low dose survived challenge infection compared to 75% survival in mice with a high vaccine dose, suggesting the preexisting immunity could be overcome by increased vaccine dose.

## Poxvirus vectors

Viral vectors work as platform technologies for several important pathogens, and their development is important for Biodefense vaccines inducing protective immunity against multiple pathogens with limited investment. Most types of vector vaccines are very safe due to their highly attenuated backbone. Poxvirus vector is heat-stable and suitable for long-term storage of vaccine, and allows stable insertion of one or more foreign genes (Henderson and Moss, 1999). The WR strain of vaccinia virus (genus orthopoxvirus) is neurovirulent (Buller et al., 1985). It was modified to express RVFV Gn and Gc in place of thymidine kinase (TK), and found to be highly attenuated (Collett et al., 1987). Papin et al. used attenuated recombinant Copenhagen strain (vCO) which lacks IFN-γ receptor homolog gene (B8R) and encodes TK gene inactivated by the expression of RVFV Gn and Gc (vCOGnGc) or Gn, Gc and human IFN-γ (vCOGnGcγ) in place of TK (Papin et al., 2011). Severe combined immunodeficiency (SCID) mice vaccinated (i.p.) with 1 × 10^7^ pfu of either vaccines did not manifest weight loss nor death, suggesting their safety. CB6F_1_ mice immunized (i.m.) twice with vCOGnGc or vCOGnGcγ were challenged with ZH501 strain resulting in 90 or 60% protection, respectively, while single dose vaccination conferred only 50 or 10% protection, respectively (Table 1). *Papio cynocephalus anubis* baboons vaccinated with vCOGnGc by scarification at day 0 (1 × 10^7^ pfu), and i.m. booster at day 28 (1 × 10^9^ pfu) induced PRNT_80_ 1:512 or more, while vaccination with a single dose induced PRNT_80_ 1:16 to 1:64 by day 28. Thus, vCOGnGc will be a safe and heat-stable candidate RVF vaccine for humans.

An attenuated Lumpy skin disease virus (LSDV) KS1 strain of Capripoxvirus (CPV) (genus capripoxvirus), derived from a virulent field isolate (LSDV 0240 strain), was modified as a vector for RVFV Gn and Gc (rLSDV-RVFV or rKS1/RVFV) (Wallace et al., 2006; Soi et al., 2010). LSD is endemic to Africa, while the vector has restricted host range to ruminants, and confers potential cross-protection from sheep pox or goat pox in ruminants. Sheep vaccinated twice with 1 × 10^6^ or 1 × 10^7^ pfu of rLSDV-RVFV (i.d.) did not show an increase in rectal temperature after challenge with wt RVFV or sheep poxvirus (Table 1) (Wallace et al., 2006). On the other hand, sheep vaccinated twice with other similar CPV vector vaccine, rKS1/RVFV (s.c.), and challenged with wt RVFV did not induce viremia, while those inoculated with saline or rKS lacking RVFV Gn/Gc induced 10^1^–10^4^ TCID_50_/ml of viremia (Soi et al., 2010). Also, sera derived from those sheep vaccinated with rKS1/RVFV neutralized both RVFV and sheep poxvirus, and no vaccinated sheep developed fever or skin lesions after sheep poxvirus challenge. Effect of preexisting antibodies against sheep poxvirus was also evaluated (Soi et al., 2010). Sheep challenged with sheep poxvirus 1 month before vaccination with rKS1/RVFV raised detectable neutralizing antibodies only after booster, while all vaccinated sheep did not show viremia after RVFV challenge. Those LSDV vaccines could also confer protective immunity in mice regardless of host range of CPV (Wallace et al., 2006; Soi et al., 2010; Ayari-Fakhfakh et al., 2012). Although LSDV is classified as a select agent by USDA, the vaccine will be suitable for dual vaccination of ruminants for CPV diseases and RVF in endemic countries.

## Newcastle disease virus vectors

Newcastle disease virus (NDV) (genus *Avulavirus*, family *Paramyxoviridae*) causes diseases in all birds, and three pathotypes are known; lentogenic (no disease), mesogenic (intermediate), and velogenic (highly virulent) (Huang et al., 2003). The non-segmented negative-stranded RNA (15,186 nt.) genome has six structural genes; 5′-NP-P-M-F-HN-L-5′. Lentogenic LaSota strain has been used as a live-attenuated vaccine for NDV in poultry, and the recombinant LaSota strain encoding foreign genes was developed for vaccine use by using reverse genetics (Peeters et al., 1999; Romer-Oberdorfer et al., 1999; Huang et al., 2001). The virus is non-pathogenic to mammals, the V protein encodes host-specific IFN antagonist activity (Park et al., 2003), and there is less chance of any pre-existing immunity to the virus because mammals are not the natural hosts of NDV. In addition, NDV-based vaccine can be produced in embryonated eggs, a technology that is already available for influenza vaccine production and easily scalable for industrial production.

NDV-based vaccine is considered as a veterinary vaccine candidate for RVF. Recombinant NDV LaSota strain expressing RVFV Gn in place of NDV M (matrix protein) (NDFL-Gn) was generated and characterized (Kortekaas et al., 2010a). Cattle vaccinated twice with NDVFL-Gn vaccine developed 1:8–1:32 of neutralizing antibodies (Cytopathic Effect Neutralization Test, CPENT: endpoint of serum dilution with 100% inhibition of cytopathic effect) (Swanepoel et al., 1986) by i.m. route but not by i.n. route. NDFL2-GnGc which encodes both Gn and Gc was also generated, and produced virus-like particles consisting of Gn and Gc in allantoic fluid in embryonated eggs (sucrose density 1.14–1.15 g/cm^3^) distinct from that of paramyxovirus (1.18–1.20 g/cm^3^), while it remains unknown whether the NDFL2-GnGc incorporated Gn and Gc into NDV virions (Kortekaas et al., 2010b). All the Balb/c mice vaccinated twice (i.m.) with 1 × 10^5.3^ TCID_50_ of NDFL2-GnGc and challenged with wt RVFV strain survived (Table 1) without the increase in anti-N antibody suggesting the vaccination induced sterile immunity. Lambs vaccinated twice (i.m.) with 1 × 10^7.3^ TCID_50_ of NDFL2-GnGc developed more than 1:100 of neutralizing antibody (CPENT) (Kortekaas et al., 2012). Similar neutralizing response induced by i.m. route was obtained by s.c., while i.n. administration did not elicit neutralizing antibody responses in sheep (Harmsen et al., 2011). In the subsequent study, lambs vaccinated once (i.m.) with 1 × 10^7^ TCID_50_ of NDFL2-GnGc (NDV-GnGc) and challenged with wt RVFV at day 19 post-vaccination showed reduced viremia without significant change in rectal temperature (Table 1), whereas neutralizing antibody was developed only in 33% before challenge (Kortekaas et al., 2012).

## Concluding remarks

Next generation vaccines are probably superior to traditional vaccines in safety or immunogenicity. However, the information of safety and immunogenicity is still limited to specific animal models. It is unclear how the variety of assay procedures (e.g., route, dose, virus/host strains, antibody detection method, etc.) by different laboratories affects the interpretation of their efficacy results. Most vaccine candidates will further require rigorous safety and efficacy testing using appropriate animal models before human clinical trials. The correlation of neutralizing antibody level with prevention of viremia and clinical signs should be analyzed in mouse models, ruminants, and non-human primates for those novel vaccine candidates in the future.

### Conflict of interest statement

The authors declare that the research was conducted in the absence of any commercial or financial relationships that could be construed as a potential conflict of interest.
